# The self-activated radical doping effects on the catalyzed surface of amorphous metal oxide films

**DOI:** 10.1038/s41598-017-12818-1

**Published:** 2017-09-29

**Authors:** Hong Jae Kim, Young Jun Tak, Sung Pyo Park, Jae Won Na, Yeong-gyu Kim, Seonghwan Hong, Pyeong Hun Kim, Geon Tae Kim, Byeong Koo Kim, Hyun Jae Kim

**Affiliations:** 1 School of Electrical and Electronic Engineering, Yonsei University, 50 Yonsei-ro, Seodaemun-gu, Seoul, 120-749 Republic of Korea; 2 LG Display Co., Ltd., 1007, Deogeun-ri, Wollong-myeon, Paju-si, Gyeonggi-do 413-811 Republic of Korea

## Abstract

In this study, we propose a self-activated radical doping (SRD) method on the catalyzed surface of amorphous oxide film that can improve both the electrical characteristics and the stability of amorphous oxide films through oxidizing oxygen vacancy using hydroxyl radical which is a strong oxidizer. This SRD method, which uses UV irradiation and thermal hydrogen peroxide solution treatment, effectively decreased the amount of oxygen vacancies and facilitated self-passivation and doping effect by radical reaction with photo-activated oxygen defects. As a result, the SRD-treated amorphous indium-gallium-zinc oxide (a-IGZO) thin film transistors (TFTs) showed superior electrical performances compared with non-treated a-IGZO TFTs. The mobility increased from 9.1 to 17.5 cm^2^/Vs, on-off ratio increased from 8.9 × 10^7^ to 7.96 × 10^9^, and the threshold voltage shift of negative bias-illumination stress for 3600 secs under 5700 lux of white LED and negative bias-temperature stress at 50 °C decreased from 9.6 V to 4.6 V and from 2.4 V to 0.4 V, respectively.

## Introduction

Research on amorphous oxide-based semiconductors (AOSs) have attracted attentions as a leading candidate for flexibility, large scale, and transparent electrical devices due to high mobility, high optical transparency, and low temperature deposition compared to amorphous silicon^1–3^. Recently, these AOSs were used in various flexible devices^4^ and a sensor array^5^ as the active layer. However, the AOSs devices still suffer from instability issues such as illumination, bias, and temperature stress. In general, the origin of the instability of AOSs comes from carrier trapping and injection, ambient gas interaction, and oxygen vacancy (V_0_)^6–9^. The fundamental studies regarding first and second origins showed that the instability can be improved using a high quality gate dielectric and an appropriate passivation layer, respectively^10,11^. On the other hand, the instability problem related to V_0_ is difficult to address because V_0_ is involved in the intrinsic property of AOSs. To solve this issue, AOSs are deposited under the oxygen-rich condition in order to reduce oxygen vacancy, but it inevitably accompanies a decrease in mobility. Thus, many studies regarding the reduction of V_o_ without mobility deterioration, such as UV annealing^12^ and high pressure oxygen annealing^13^, are reported. These methods can increase the metal oxide bond and decrease V_o_ by chemical oxidation but it requires a prolonged time (1–2 hours) and additional external energy source. These studies are relatively difficult to apply in the manufacturing process because a huge amount of time and expensive equipments are required for them to be implemented on the large-scale substrates. Thus, it is necessary to develop an effective, largely practical, and simple method to improve the stability of AOSs. In this respect, the strong oxidizer, which is a hydroxyl radical, can preferentially react with oxygen defects without prolonged treatment and an additional energy source. The hydroxyl radical (OH*) obtained from the decomposition of hydrogen peroxide (H_2_O_2_), is generally used to eliminate organic components in the solution process due to its high oxidation potential compared to oxygen and ozone^14–16^. According to the previous reports, the H_2_O_2_ is decomposed to OH* by photolysis^17^, pyrolysis^18^, catalytic pyrolysis, and catalysis on the surface of the metal oxide particle^19,20^. In the case of catalytic pyrolysis and catalysis, the decomposition rate depends on the surface area of the oxide particles. This decomposition behavior in catalytic pyrolysis and catalysis has been taken into account for this defective oxide surface because the increase in surface area is accompanied by an increase in the number of defect sites on the surface. In general, this defective oxide surface, induced by UV irradiation, is called “hydrophilic surface”. According to the Sun, R. –D. *et al*.^21^, the degree of the defective oxide surface is proportional to the degree of the hydrophilic surface which is defined as the contact angle. Therefore, the strong oxidation of OH* can be used on the oxide surface by appropriate UV irradiation and thermal H_2_O_2_ solution treatment. In addition, the UV irradiation can also lead to the transition of V_0_ to V_0_^2+^ states as releasing the free electrons in the oxide films and this V_0_^2+^ tends to get back to the ground state after a certain amount of time^22^. Normally, these metastable defects cause persistent photoconductivity (PPC) and negative bias-illumination stress (NBIS) degradation in most of the AOSs^22,23^. In this respect, these metastable oxygen defects can easily react with OH*. On the basis of these background examples, we suggest the SRD method using the spontaneous decomposition of the H_2_O_2_ solution and radical reaction on the highly hydrophilic oxide surface to decrease defects related to oxygen in AOSs film. We also investigated this SRD effect for radical oxidation through electrical characteristics and stability, and conducted chemical analysis using X-ray photoelectron spectroscopy (XPS) and Fourier transform infrared spectroscopy (FTIR).

## Results and Discussion

**Figure 1:**
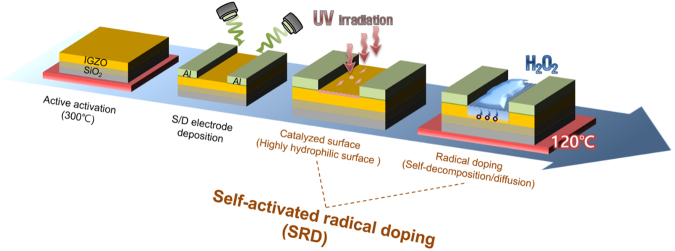
Schematic illustration of SRD a-IGZO TFTs process.

**Figure 2:**
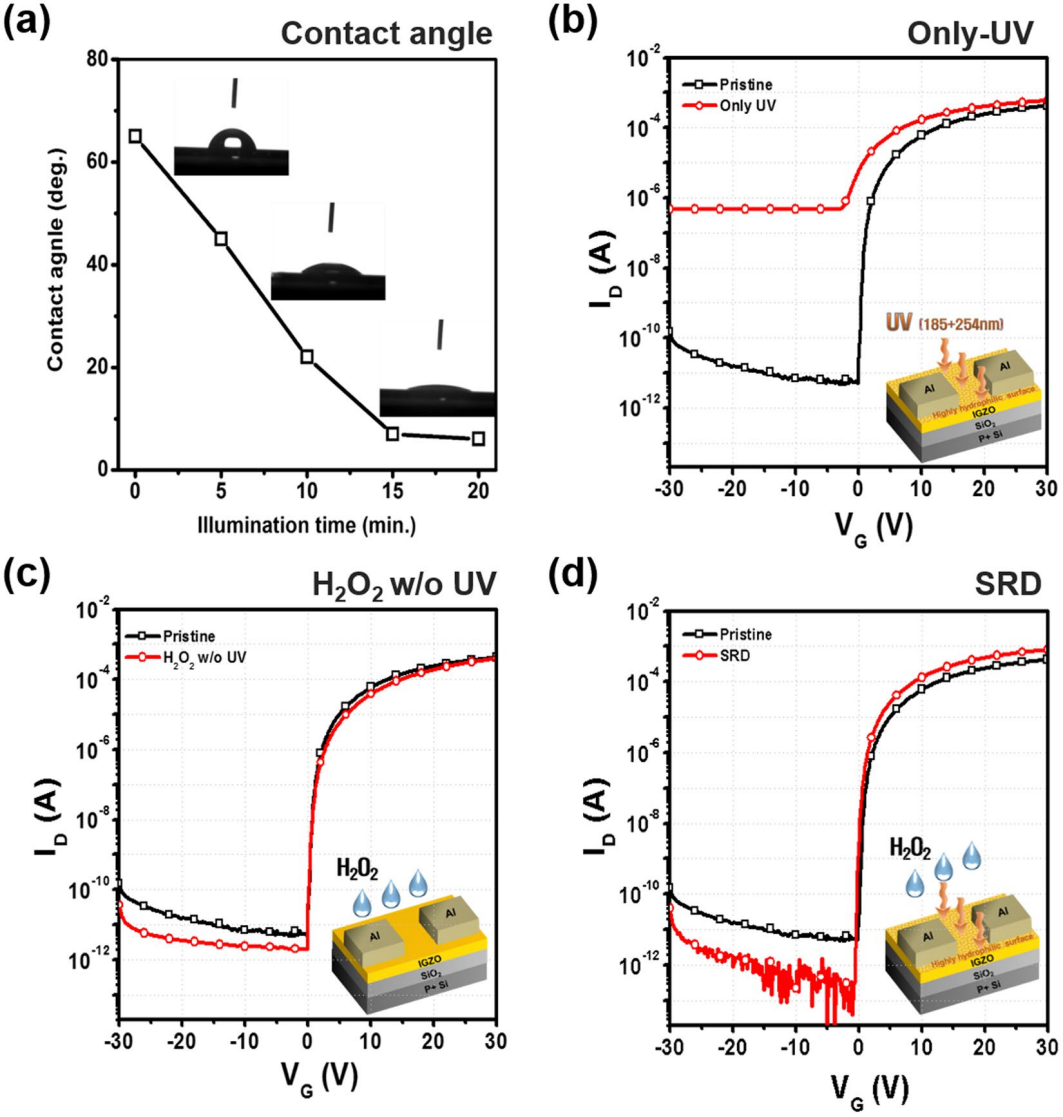
Variations of (**a**) contact angle of a-IGZO surface with illumination time and transfer characteristics with different conditions: (**b**) Only-UV, (**c**) SRD, (**d**) H_2_O_2_ without UV irradiation.

Figure 1 shows the overall process adapted for the fabrication of SRD a-IGZO TFTs. The SRD was carried out by three steps that are comprised of UV irradiation to generate a defective surface (highly hydrophilic surface), H_2_O_2_ treatment for radical doping, and thermal annealing. First of all, it is necessary to verify the highly hydrophilic surface for radical generation. According to Sun, R. –D. *et al*.^21^, the contact angle represents the degree of hydrophilic and it depends on the UV irradiation time. Then, we measured the contact angle of the a-IGZO surface as increasing UV irradiation time in order to investigate the minimal time for the highly hydrophilic surface. Figure 2(a) shows that the UV irradiated a-IGZO surface has 7° in a UV illumination time of 15 mins. This is similar to the super-hydrophilic surface condition, which is defined as being under 5°. These results showed that the defective a-IGZO surface was formed by our UV irradiated condition.

Figure 2(b–d) shows transfer characteristics with different treatment conditions, including only-UV, H_2_O_2_ without UV and SRD. First, the only-UV treated device exhibits a large increase in off current, as shown in Fig. 2(b). This is because UV irradiation causes the generation of electron-hole pairs, ionization of V_o_, and photo-reduction of weak chemical bonds in the a-IGZO films. Second, in the H_2_O_2_ without UV samples, non-UV treated IGZO surface is a strictly hydrophilic surface, which is defined as being under 90°, in the above contact results. However, as shown in Fig. 2(c), there is no remarkable change of transfer characteristics in the only H_2_O_2_ treated device without UV irradiation. On the other hand, the SRD a-IGZO TFTs distinctly exhibit much improved transfer characteristics compared to other treated devices, as shown in Fig. 2(d). These results indicated that the combined UV irradiation and H_2_O_2_ treatment are essential for improving electrical characteristics. Table 1 summarizes the electrical parameters of a-IGZO TFTs with different treatments, including field-effect mobility (μ_FET_), on/off ratio, and sub-threshold swing (S.S).

**Table 1:** Summary of the electrical parameters including μ_FET_, on/off ratio, and S.S for different treatments.

Sample	μ_FET_ (cm^2^/V∙s)	On/off ratio	S.S (V/decade)
Non-treated	9.09	8.9 × 10^7^	0.40
Only-UV	12.93	1.25 × 10^3^	2.24
CRD	16.4	7.96 × 10^9^	0.34
H_2_O_2_ w/o UV	7.62	1.83 × 10^8^	0.39

**Figure 3:**
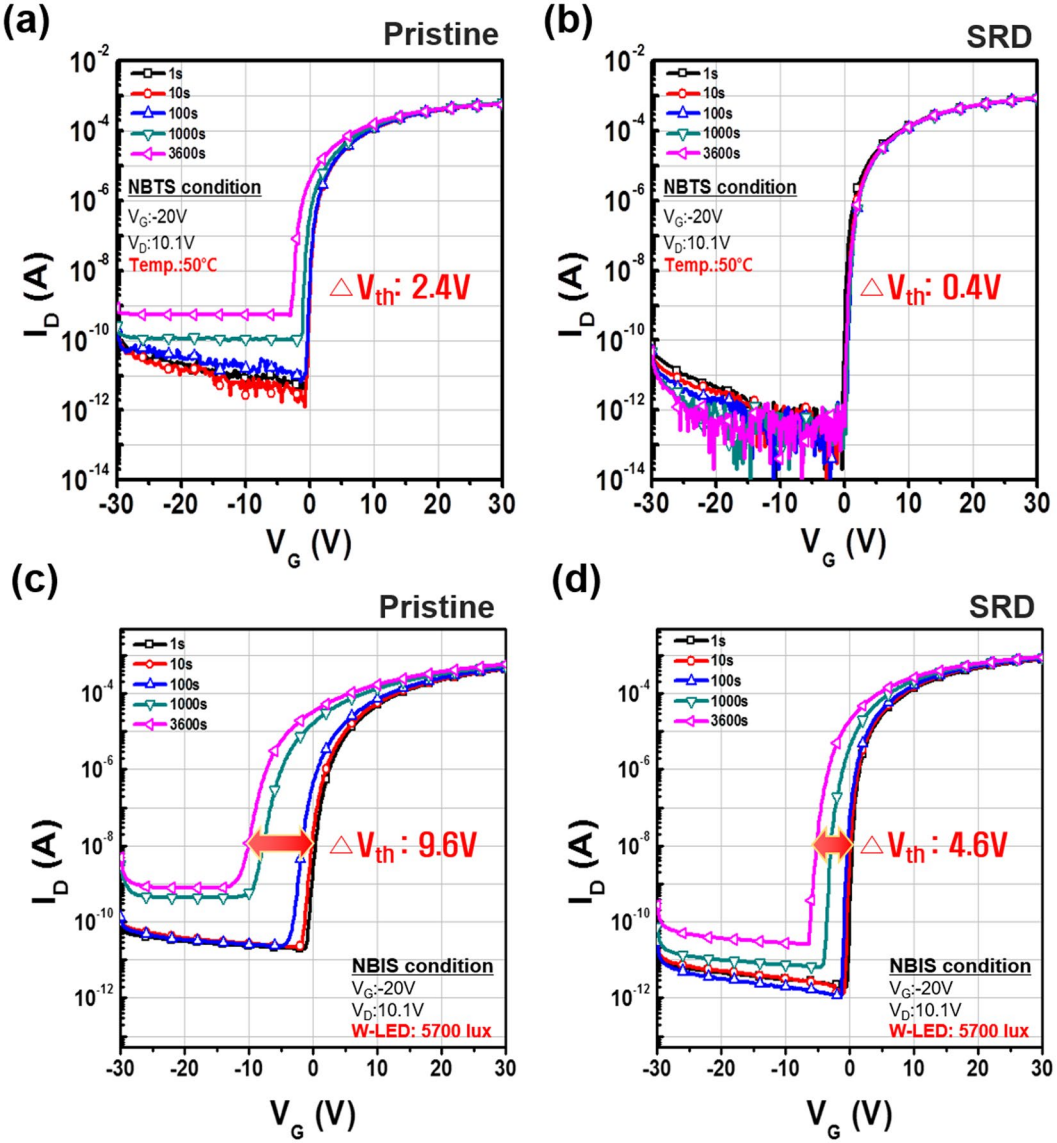
Variations of negative V_th_ shift under NBTS and NBIS stability of SRD a-IGZO TFTs with stress times: (**a**) NBTS and (**c**) NBIS stability of pristine, (**b**) NBTS and (**d**) NBIS stability of SRD a-IGZO TFTs.

The stability test is crucial for estimating structural defect sites related to V_o_ in amorphous oxide films. In particular, hydrogen in incorporated OH*, and V_o_ is associated with stability degradation in NBTS and NBIS tests^24,25^. Accordingly, the NBTS test was first performed in Fig. 3(a) and (b), showing that the V_th_ of the SRD device rarely shifts (0.4 V) whereas that of the pristine device shifts 2.4 V with increased off current. This result indicated that the hydrogen incorporated OH* does not work as interstitial states which cause to negative V_th_ shift^26^. To further examine the SRD effect regarding V_o_, we evaluated the NBIS test. The V_th_ shift of the non-treated device and SRD device are 4.6 V and 9.6 V, respectively, as shown in Fig. 3(c) and (d). Figures S1 and S2 show the results of NBTS and NBTIS tests for non-treated and SRD treated devices for 10000 s at 80 and 100°C, respectively. The V_th_ shift of the SRD device is much less than that of non-treated device. This result showed that the SRD method is effective in improving the stability of NBTS and NBIS caused by interstitial atom and oxygen vacancy ionization. Additionally, we conducted a PBS test. As shown in Figure S3 (a) and (b), the V_th_ shift of the SRD device is less than that of the pristine. This result also showed that the SRD method can enhance the stability of PBS is originated from carrier trapping caused by oxygen defects and ambient gas interaction in the non-passivation oxide device^10,27^. We also re-measured the electrical performance of the SRD a-IGZO TFTs after 3 months because it is possible that they would be affected by the photo-generated carrier recombination and ionized V_o_ neutralization. As shown in Figure S4, the enhanced transfer characteristics of SRD a-IGZO TFTs were retained even after 3 months. Therefore, these outstanding stability results have significant implications for a explaining the SRD effect, which can decrease the defect sites that are related to V_o_. This is not a temporary effect.

**Figure 4:**
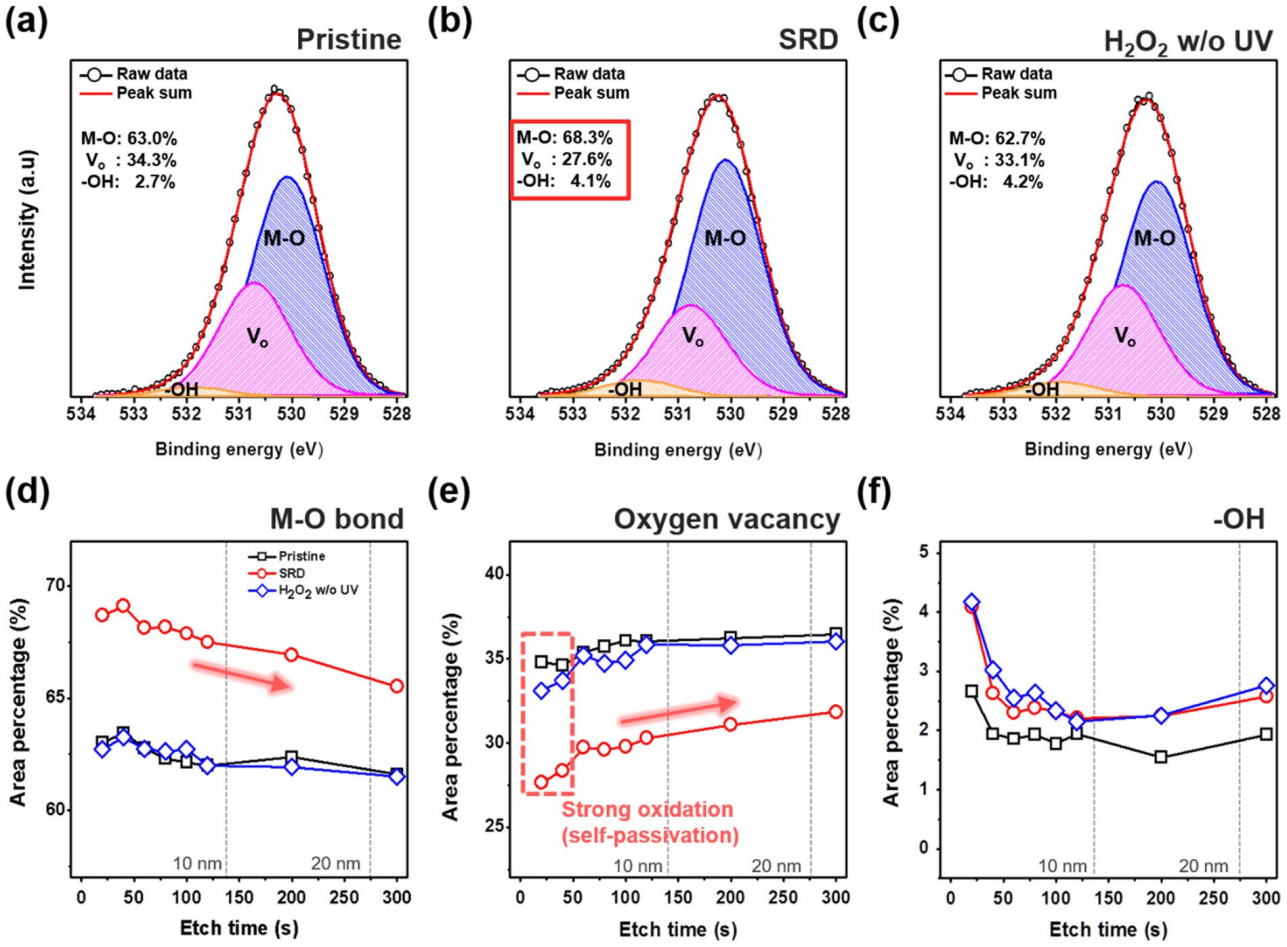
XPS results from deconvolution of O 1 s spectra for the a-IGZO film under different conditions: (**a**) Pristine (**b**) SRD, (**c**) H_2_O_2_ w/o UV at 20 secs etched region, and area percentage of XPS depth profile with etch time: Variations of (**d**) M-O, (**e**) V_o_, (**f**) -OH.

**Figure 5:**
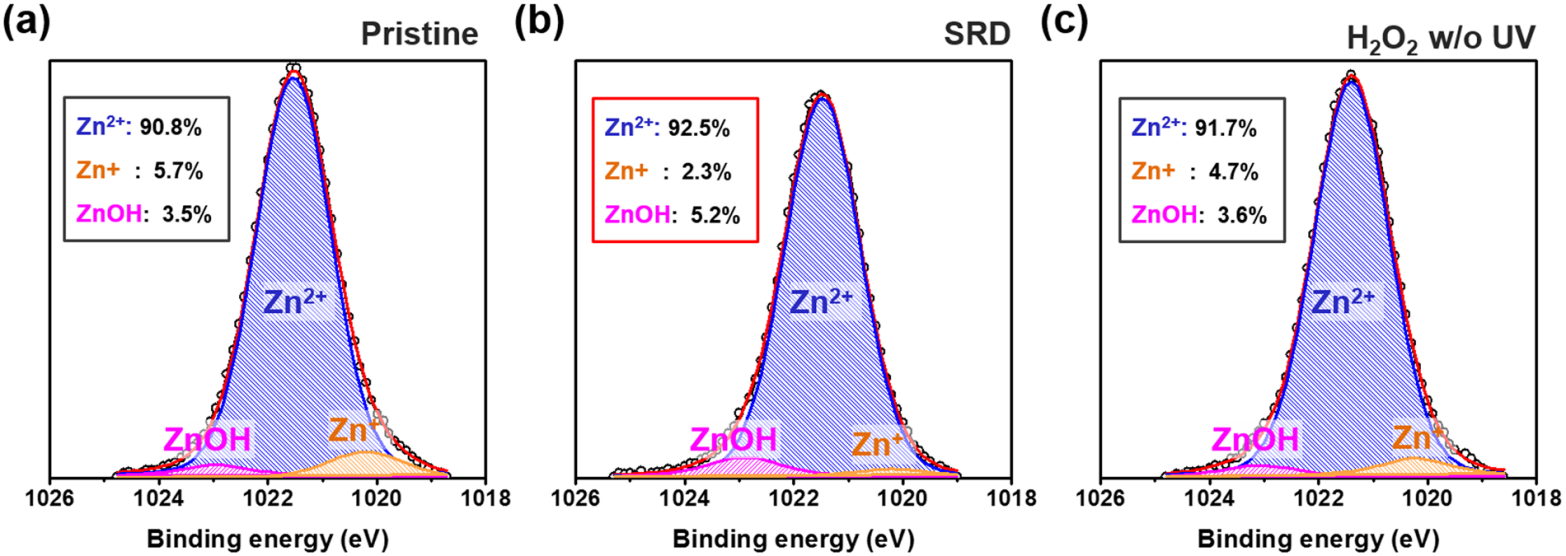
Zn 2p3/2 XPS spectra of a-IGZO film with different conditions: (**a**) Pristine, (**b**) SRD, and (**c**) H_2_O_2_ w/o UV at 20 secs etched region.

We performed an XPS depth analysis to confirm the chemical characteristics and the SRD effect. Figure 4 shows the O1s spectra for a-IGZO film with a different treatment condition. The O1s spectra were deconvoluted into three different peaks that were centered at 530.1 ± 0.2, 531.0 ± 0.2, and 532.0 ± 0.2 eV. The first peak represents lattice oxygen with a low binding energy related to In, Zn, and Ga metal-oxide (M-O) bonds. The second peak with middle binding energy and the high binding energy peak correspond to V_o_ and weakly bonded hydroxyl groups (-OH), respectively^28^. Figure 4(a–c) show that the SRD a-IGZO films have a higher M-O bond and the lowest oxygen vacancies compared with those of non-treated and H_2_O_2_ w/o UV treated devices. This SRD effect was maintained with film depth, as shown in Figure (d–f). In addition, it was found that the oxygen vacancies rapidly decrease in the area near the back-channel region by the radical oxidation, as shown in Fig. 4(e). These findings appear to demonstrate the reasons for the improved stability results by the decrease in V_0_ and reduced V_0_ layer effect, which is similar to the self-passivation effect, at the back-channel surface region^29^. Additionally, to investigate the OH* bond with metal cation, we analyzed Zn 2p3/2 XPS spectra because Zn atoms can react well with oxygen species compared to In and Ga, due to their lower bond energy^30^. As a result, Fig. 5 shows that the SRD a-IGZO films have an increase in Zn-O (Zn^2+^) and Zn-OH bond, and a decrease in Zn interstitials (Zn^+^) compared to those of the pristine and H_2_O_2_ without UV treated device. This means that OH* has preferentially bonded with V_0_ and metal cation defects in a-IGZO films for the increase in Zn-O (Zn^2+^) and decrease in Zn^+^. We additionally investigated the location of the Fermi energy level of SRD a-IGZO films in valence band offsets derived from XPS data to verify the reason for the improvement of μ_FET_ despite the decreasing V_0_ concentration^31,32^. For the SRD a-IGZO films, the Fermi level was located relatively close to the conduction band, as shown in Figure S5. The Fermi energy level shift can be explained by the change in carrier concentration.

**Figure 6:**
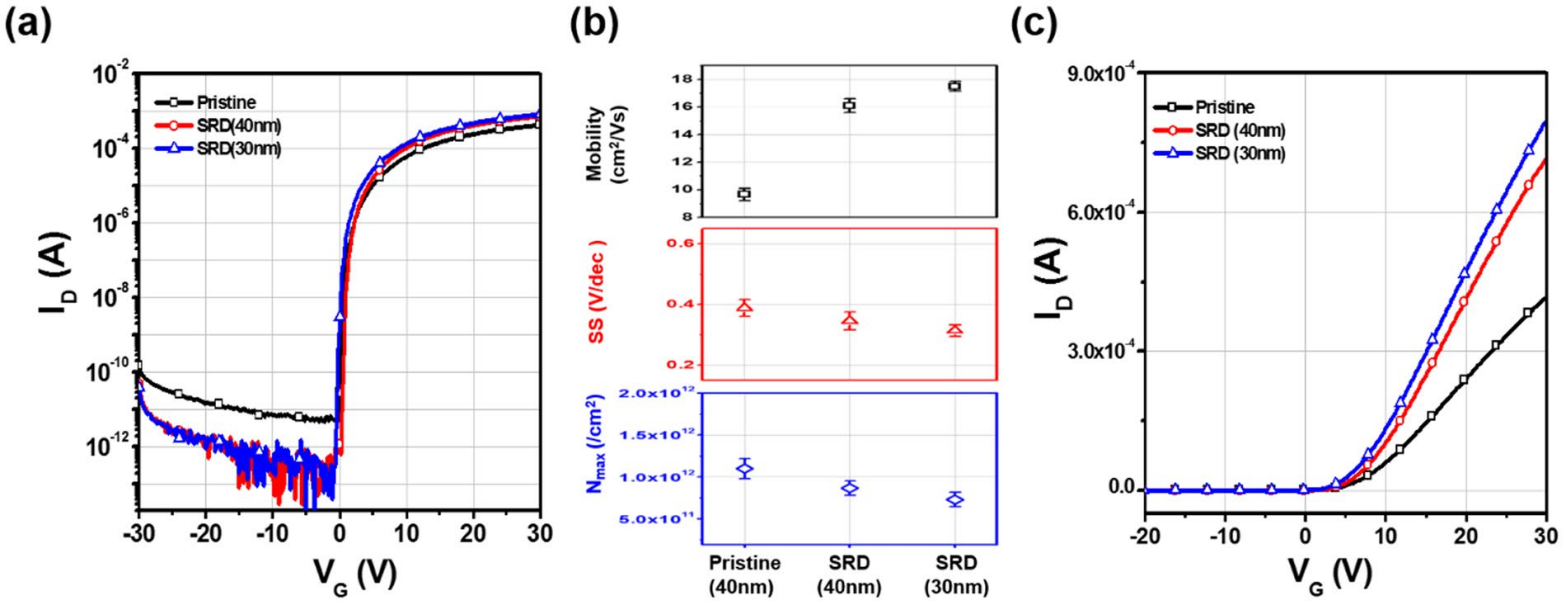
Transfer characteristics of pristine and SRD a-IGZO TFTs with decreasing channel thickness: (**a**) log scale, (**b**) statistical parameters including μ_FET_, S.S, and N_max_, and (**c**) Linear scale.

As shown in the XPS depth results, the SRD can affect a-IGZO films with depth. In order to confirm the SRD effect, we re-investigated the electrical transfer characteristics with decreasing channel thickness for the reduction of the radical inter-diffusion distance from the back-channel surface. Figure 6 shows the transfer characteristics with decreasing channel thickness and statistical parameters, including μ_FET_, S.S, and maximum trapped charge density (N_max_). N_max_ was extracted from the transfer characteristics using the following relationship:

1 $${N}_{\max }=(\frac{S.S\,\bullet \,\mathrm{log}(e)}{kT/q}-1)\frac{{C}_{i}}{q}$$

where k is the Boltzmann constant, T is the absolute temperature, C_i_ is the gate capacitance per unit area, and q is the elementary charge. The SRD, the thinner a-IGZO TFTs, have a superior transfer characteristic. The μ_FET_ is additionally improved from 16.1 to 17.5 cm^2^/V•s, S.S is decreased from 0.35 to 0.32 V/decade, and N_max_ is decreased from 8.67 × 10^11^ to 7.3 × 10^11^ cm^−2^.

It can be demonstrated that the reduction effect of V_0_ increases in the thinner a-IGZO TFTs and it is also possible to decrease defect sites near the interface region between the channel and the gate dielectric by radical oxidation because it was closer from the back-channel to the interface region as the channel thickness decreased. Furthermore, to indirectly confirm the radical diffusion, we performed FTIR analysis with a differently treated bi-layer a-IGZO structure that was comprised of depositing sputter a-IGZO (30 nm) on the solution-processed a-IGZO, including rich-organic components. Figure S6 shows the FTIR spectra of the non-treatment, H_2_O_2_ without UV and SRD-treated bi-layer a-IGZO samples. These spectra support the diffusion of OH* during the SRD treatment because OH* is a powerful oxidizer to organic components. Thus, we can verify the diffused radical reaction through the variation of organic (C-H bond) peak in solution-processed IGZO. The C-H peak-related bending vibration at a range of 900–1370 cm^−1^ and stretching vibration at 2900–3100 cm^−1^ is evidently suppressed in the spectra of the SRD-treated sample^33^. This FTIR result shows OH* can moderately diffuse in the a-IGZO films. Therefore, these results indicated that the SRD is more effective as the channel thickness decreased and it can also promote the interface characteristics of a-IGZO films.

**Figure 7:**
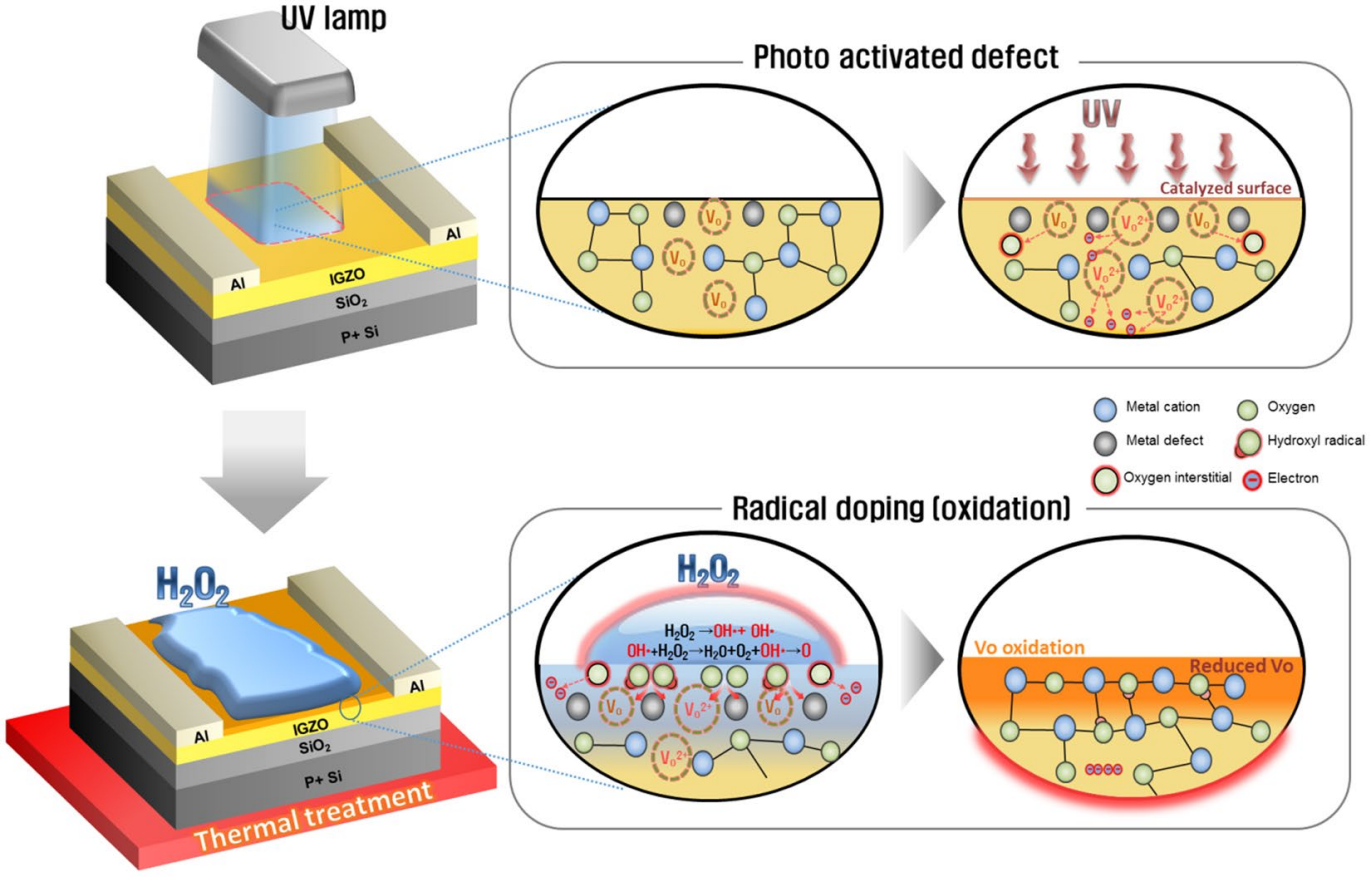
The schematic illustration of SRD mechanism.

We propose the chemical mechanism of SRD, as illustrated in Fig. 7. On the basis of the above analysis data, the SRD mechanism can be demonstrated by photo-activated defects in the surface region and radical oxidation by thermally radical solution treatment, as shown in Fig. 7. The energies of UV light at 185 nm and 254 nm are 6.7 eV and 4.8 eV, respectively. This UV energy is greater than the binding energy of O_2_ (5.13 eV) as well as the binding energy of In-O (1.7 eV), Ga-O (2.0 eV) and Zn-O (1.5 eV)^34,35^. Thus, the UV light can make defect sites related to oxygen vacancies, oxygen interstitials, and interstitial metal cations in the oxide surface region by breaking the bond, and the following equations (2) and (3) generally agreed with the decomposition reaction in H_2_O_2_ kinetics according to the previous reports^36,37^.

2 $${{\rm{H}}}_{2}{{\rm{O}}}_{2}+{\rm{defect}}\,{\rm{sites}}\to {{\rm{OH}}}^{\ast }+{{\rm{OH}}}^{\ast }$$

3 $${{\rm{H}}}_{2}{{\rm{O}}}_{2}+{{\rm{OH}}}^{\ast }\to {{\rm{HO}}}_{2}^{\ast }\,+{{\rm{OH}}}^{\ast }\to {{\rm{H}}}_{2}{\rm{O}}+{{\rm{O}}}_{2}$$

The initial step of the catalytic decomposition of H_2_O_2_ on an amorphous oxide surface is homolytic cleavage of the O-O bond to form two OH*^19^. The further reaction of OH* with H_2_O_2_ rises to form the oxygen molecule at the surface region. This decomposition behavior would also be promoted by a thermally treated H_2_O_2_ solution (80 °C) because the activation energy of H_2_O_2_ decomposition decreases and the decomposition rate increases in accordance with an increase in the H_2_O_2_ solution temperature^20^. In addition, the oxygen atom can be partially generated through a second reaction of oxygen molecules and ${{\rm{OH}}}^{\ast }({{\rm{OH}}}^{\ast }+{{\rm{O}}}_{2}\to {{\rm{HO}}}_{2}^{\ast }+O)$ ^38^. These generated radical and oxygen atoms under thermal annealing, can preferentially bond with oxygen and metal cation defects, including ionized V_o_, metal cation interstitial, and oxygen interstitial. In other words, the UV irradiation artificially ionizes V_0_ in deep level states, and the generated OH* through thermal radical solution treatment on the catalytic surface can make a chemical reaction with these metastable defects (ionized V_o_). Moreover, this radical has a high reactivity and diffusivity, thus, it would vigorously react at the back-channel surface of a-IGZO film and this reaction would also lead to the formation of a reduced V_0_ layer at the back-channel surface region. This reduced V_0_ layer inhibits the interaction of the adsorption of molecules from ambient air^39^. Additionally, some diffused radical can also decrease oxygen defects near the interface region of the a-IGZO films and gate dielectric. Lastly, the μ_FET_ improvement effect can be taken into account for the change of carrier concentration as well as the decrease in interface trap sites. The change of carrier concentration can be brought about by the reaction of H_2_O_2_ and oxygen interstitials (O_i_) generated by UV irradiation^37^. Therefore, the SRD method facilitates an effective decrease in artificially activated V_0_, an increase of self-passivation effect, and improvement of μ_FET_ by radical oxidation through sequential UV and thermal radical solution treatment.

## Conclusions

In this study, we investigated self-activated radical doping (SRD), which consists of UV irradiation and thermal radical solution treatment. The SRD a-IGZO TFTs exhibited remarkably improved electrical performances. The μ_FET_ and on/off ratio were improved from 9.1 to 17.5 cm^2^/Vs and from 8.9 × 10^7^ to 7.96 × 10^9^, respectively. The negative and positive V_th_ shift evidently decreased under bias, temperature, and illumination stress and the enhanced transfer characteristics were retained after 3 months. In addition, we confirmed that the SRD mechanism demonstrated radical oxidation with defects related to oxygen from XPS depth and FTIR analysis. Therefore, we concluded that the SRD method can effectively decrease V_0_ and induce self-passivation at the back-channel surface of a-IGZO film. Moreover, the SRD showed it is possible to control not only the V_o_ but also the interface property of amorphous oxide and gate dielectric using the non-vacuum method.

## Methods

### Fabrication of thin film transistors (TFTs)

First, we fabricated the bottom-gate a-IGZO TFTs structure by depositing a-IGZO on the heavily doped p^+^-Si wafer with thermally oxidized SiO_2_ of 120 nm. Then, 40 nm a-IGZO was deposited by RF magnetron sputtering as the active layer using 3-inch IGZO target (In_2_O_3_:Ga_2_O_3_:ZnO = 1:1:1 mol%) under rf power of 150 W, working pressure of 5 mTorr, and total deposition time of 5 mins at room temperature. Following the active layer deposition, the samples were annealed at 300 °C for 1 hour in ambient air. And then, 100 nm Al source and drain electrodes were deposited by a radio frequency sputter system with a shadow mask. The channel region of TFTs was defined with a width of 1000 μm and a length of 150 μm.

### The SRD treatments

The SRD was performed on a-IGZO TFTs by a three-step process as shown in Fig. 1. First, these samples were irradiated for 15 mins by mercury lamp-based UV light, where there was a wavelength of 185 nm and 256 nm and a photon flux density of 60 mW/cm^2^. Then, these samples were dipped in the 30% H_2_O_2_ (80 °C) solution for 20 secs. Finally, these samples were thermally annealed at 120 °C for 5 mins. We also fabricated only-UV and H_2_O_2_ without UV treated samples for verification of the SRD effect.

### Electrical and chemical measurements

The electrical characteristics of the SRD TFTs were measured in the dark at room temperature using an HP4156C semiconductor parameter analyzer. To evaluate negative bias- temperature stress (NBTS) and NBIS stability, V_GS_ = −20 V and V_DS_ = 10.1 V at 50 °C, and under 5700 lux of white LED were applied for 3600 secs in air, respectively. Moreover, positive bias-stress (PBS) at V_GS_ = 20 V and V_DS_ = 10.1 V were applied for 3600 secs in ambient air. To investigate the chemical characteristics after SRD, we performed a depth profile XPS (Thermo Scientific. K-alpha) and Attenuated Total Reflection FTIR (ATR-FTIR, Bruker Vertex 70). XPS analyses were used to monitor the variation of composition, chemical structure, and valence band offset in oxide films.

### Fabrication of bi-layered a-IGZO structure for FTIR analysis

We additionally fabricated a bi-layered IGZO structure with solution-processed IGZO and sputtered IGZO on a p^+^-Si substrate with thermally oxidized SiO_2_. We prepared 0.3 M IGZO solutions with an In:Ga:Zn molar ratio of 5:1:2 by dissolving indium nitrate hydrate (In(NO_3_)_3_·*x*H_2_O), gallium nitrate hydrate (Ga(NO_3_)_3_·*x*H_2_O), and zinc acetate hydrate (Zn(CH_3_COO)_2_·2H_2_O) into 2-methoxyethanol (CH_3_OCH_2_CH_2_OH) solvent. The IGZO solutions were spin-coated at 3000 rpm for 30 secs. This coated film was baked for 20 min at 100 °C and then 30 nm-a-IGZO was deposited by sputtering with the above mentioned same condition.

## Electronic supplementary material

Supplementray info

## References

[1] Nomura K (2004). Room-temperature fabrication of transparent flexible thin-film transistors using amorphous oxide semiconductors. Nature.

[2] Ahn BD (2009). A Novel Amorphous InGaZnO Thin Film Transistor Structure without Source/Drain Layer Deposition. Jpn. J. Appl. Phys..

[3] Fortunate E (2004). Wide-bandgap high-mobility ZnO thin-film transistors produced at room temperature. Appl. Phys. Lett..

[4] Jaakko L (2017). Far-UV Annealed Inkjet-Printed In_2_O_3_*Semiconductor Layers for Thi*n Film Transistors on a Flexible Polyethylene Naphthalate Substrate. ACS Appl. Mater. Interfaces.

[5] Alexey AT (2003). Semiconducting metal oxide sensor array for the selective detection of combustion gases. Sensors and Actuators B.

[6] Suresh A (2008). Bias stress stability of indium gallium zinc oxide channel based transparent thin film transistors. Appl. Phys. Lett..

[7] Cross RBM (2006). *Investigating the* stability of zinc oxide thin film transistors. Appl. Phys. Lett.

[8] Park JS (2008). Electronic transport properties of amorphous indium-gallium-zinc oxide semiconductor upon exposure to water. Appl. Phys. Lett..

[9] Janotti A (2005). Oxygen vacancies in ZnO. Appl. Phys. Lett..

[10] Jeong JK (2008). Origin of threshold voltage instability in indium-gallium-zinc oxide thin film transistors. Appl. Phys. Lett..

[11] Ji KH (2010). Comparative study on light-induced bias stress instability of IGZO transistors with SiNx and SiO_2_ gate dielectrics. IEEE Electron Devices Lett..

[12] Tak YJ (2016). Reduction of activation temperature at 150 °C for IGZO films with improved electrical performance via UV-thermal treatment. J. Inf. Disp..

[13] Kim W-G (2016). High-pressure Gas Activation for Amorphous Indium-Gallium-Zinc-Oxide Thin-Film Transistors at 100 °C. Sci. Rep..

[14] Dai M-K (2015). Multifunctionality of Giant and long-lasting Persistent Photoconductivity: Semiconductor-Conductor Transition in Graphene Nanosheets and Amorphous InGaZnO Hybrids. ACS Photonics..

[15] Jones, C. W. *et al*. Applications of Hydrogen Peroxide and Derivatives. *Royal Society of Chemistry* (1999).

[16] Mardhiah MS (2015). Hydroxyl Radical-Assisted Decomposition and Oxidation in Solution-Processed Indium Oxide Thin-Film Transistors. J. Mater. Chem. C..

[17] Afzal A (2010). Anatoxin-a degradation by advanced oxidation processes: Vacuum-UV at 172nm, photolysis using medium pressure UV and UV/H_2_O_2_. Water Res..

[18] Andre T (1974). Mechanism of Hydrogen peroxide Pyrolysis. Can. J. Chem..

[19] Lousada CM (2012). Mechanism of H_2_O_2_ Decomposition on Transition Metal oxide Surfaces. J. Phys. Chem. C.

[20] Hiroki A (2005). Decomposition of Hydrogen Peroxide at Water-Ceramic Oxide Interfaces. J. Phys. Chem. B.

[21] Sun R–D (2001). Photoinduced Surface Wettability Conversion of ZnO and TiO_2_ Thin Films. J. Phys. Chem. B.

[22] Lee S (2015). Oxygen Defect-Induced Metastability in Oxide Semiconductors Probed by Gate Pulse Spectroscopy. Sci. Rep..

[23] Dai M-K (2015). Multifunctionality of Giant and long-lasting Persistent Photoconductivity: Semiconductor-Conductor Transition in Graphene Nanosheets and Amorphous InGaZnO Hybrids. ACS Photonics.

[24] Robertson J (2014). Light induced instability mechanism in amorphous InGaZn oxide semiconductors. Appl. Phys. Lett..

[25] Rim YS (2013). Defect reduction in photon-accelerated negative bias instability of InGaZnO thin-film transistors by high-pressure water vapor annealing. Appl. Phys. Lett..

[26] Xu L (2016). Rational Hydrogenation for Enhanced Mobility and High Reliability on ZnO-Based Thin Film Transistors: From Simulation to Experiment. ACS Appl. Mater. Interfaces.

[27] Chen W–T (2011). Oxygen-Dependent Instability and Annealing/Passivation Effects in Amorphous In-Ga-Zn-O Thin-Film Transistors. IEEE Electron Devices Lett..

[28] Kim M–G (2010). High-Performance Solution-Processed Amorphous Zinc-Indium-Tin Oxide Thin-Film Transistors. J. Am. Chem. Soc..

[29] Park JH (2014). Simple Method to Enhance Positive Bias Stress Stability of In−Ga−Zn−O Thin-Film Transistors Using a Vertically Graded Oxygen-Vacancy Active Layer. ACS Appl. Mater. Interfaces.

[30] Kim M–H (2014). Photochemical Hydrogen Doping Induced Embedded Two-Dimensional Metallic Channel Formation in InGaZnO at Room Temperature. ACS Nano.

[31] Tak YJ (2014). Enhanced Electrical Characteristics and Stability via Simultaneous Ultraviolet and Thermal Treatment of Passivated Amorphous In−Ga−Zn−O Thin-Film Transistors. ACS Appl. Mater. Interfaces.

[32] Tak YJ (2016). Activation of sputter-processed indium-gallium-zinc oxide films by simultaneous ultraviolet and thermal treatments. Sci. Rep..

[33] John RA (2016). Low-Temperature Chemical Transformations for High-Performance Solution-Processed Oxide Transistors. Chem. Mater..

[34] Kamiya T (2010). Doping and Defect Formation Energies in Amorphous Oxide Semiconductor a-InGaZnO_4_ Studied by Density Functional Theory. Phys. Status Solidi A..

[35] Aikawa S (2013). Effects of Dopants in InOx-Based Amorphous Oxide Semiconductors for Thin-Film Transistor Applications. Appl. Phys. Lett..

[36] Giamello E (1993). Evidence of Stable Hydroxyl Radicals and Other Oxygen Radical Species Generated by Interaction of Hydrogen Peroxide with Magnesium Oxide. J. Phys. Chem..

[37] Lousada CM (2010). Kinetics, Mechanism, and Activation Energy of H_2_O_2_ Decomposition on the Surface of ZrO_2_. J. Phys. Chem. C..

[38] Suh M (2000). Reactions of Hydroxyl Radicals on Titania, Silica, Alumina, and Gold Surfaces. J. Phys. Chem. B..

[39] Su W–Y (2008). Improving the property of ZnO nanorods using hydrogen peroxide solution. J. Cryst. Growth.

