# Supplementary material for: The self-activated radical doping effects on the catalyzed surface of amorphous metal oxide films
Source: Sci Rep. 2017 Sep 29;7:12469. doi: 10.1038/s41598-017-12818-1 (PMC5622114; doi:10.1038/s41598-017-12818-1)
Supplement: Supplementary file 1 — Supplementray info [file 41598_2017_12818_MOESM1_ESM.pdf]

Supplementary Information for

The self-activated radical doping effects on the catalyzed surface of amorphous metal oxide films

Hong Jae Kim[†,‡], *Young Jun Tak*[†], *Sung Pyo Park*[†], *Jae Won Na*[†], *Yeong-gyu Kim*[†], *Seonghwan Hong*[†], *Pyeong Hun Kim*[‡], *Geon Tae Kim*[‡], *Byeong Koo Kim*[‡], *Hyun Jae Kim*[*,†]

[†]*School of Electrical and Electronic Engineering, Yonsei University, 50 Yonsei-ro, Seodaemun-gu, Seoul 120-749, Republic of Korea*

[‡]*LG Display Co., Ltd., 1007, Deogeun-ri, Wollong-myeon, Paju-si, Gyeonggi-do, Korea*

Tel: 82-2-2123-5865; Fax: 82-2-2123-8123

*Corresponding author (e-mail: hjk3@yonsei.ac.kr)

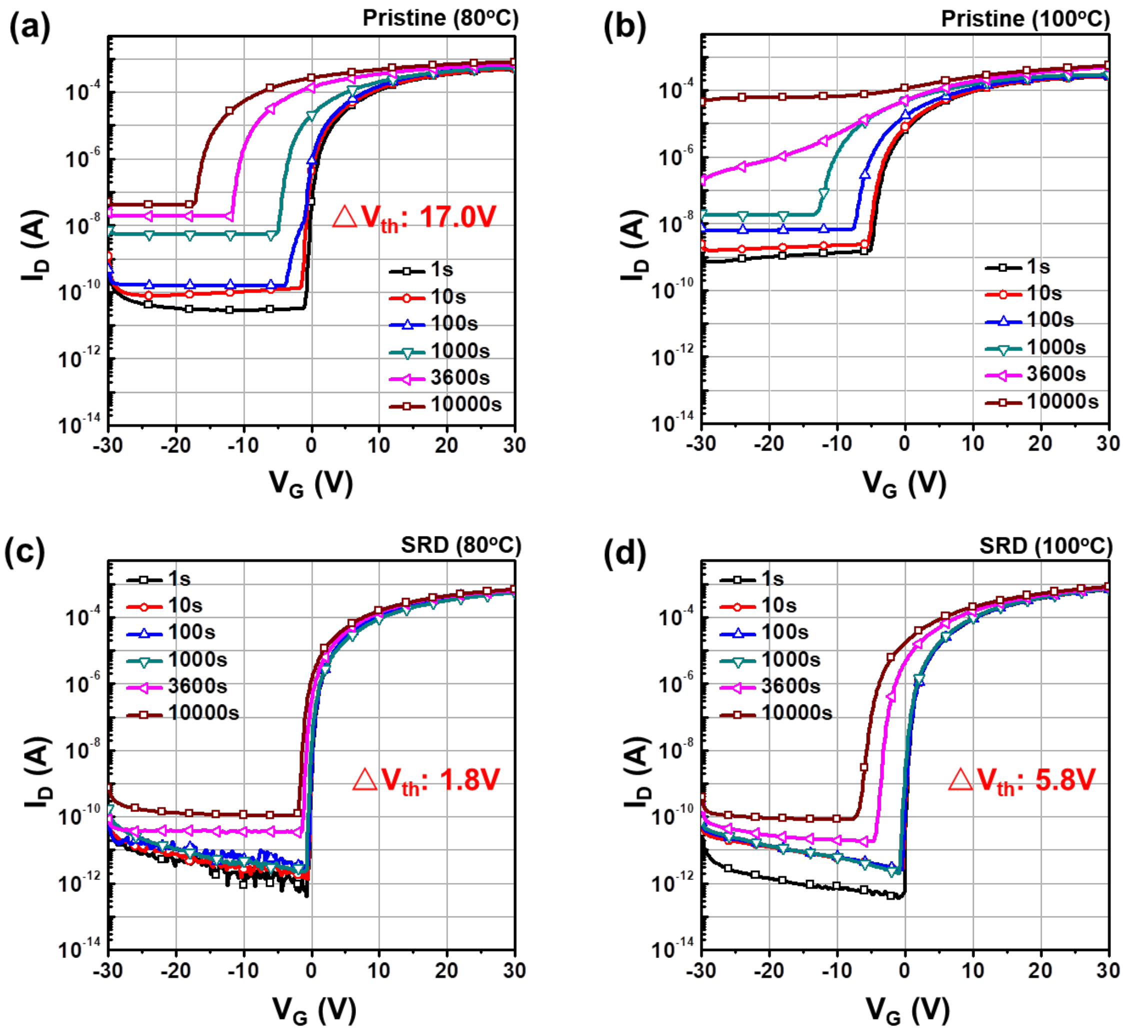

Figure S1. Variations of negative V_{th} shift under NBTS with stress times for pristine (a) at 80°C and (b) at 100°C and SRD treated IGZO TFTs (c) at 80°C and (d) at 100°C

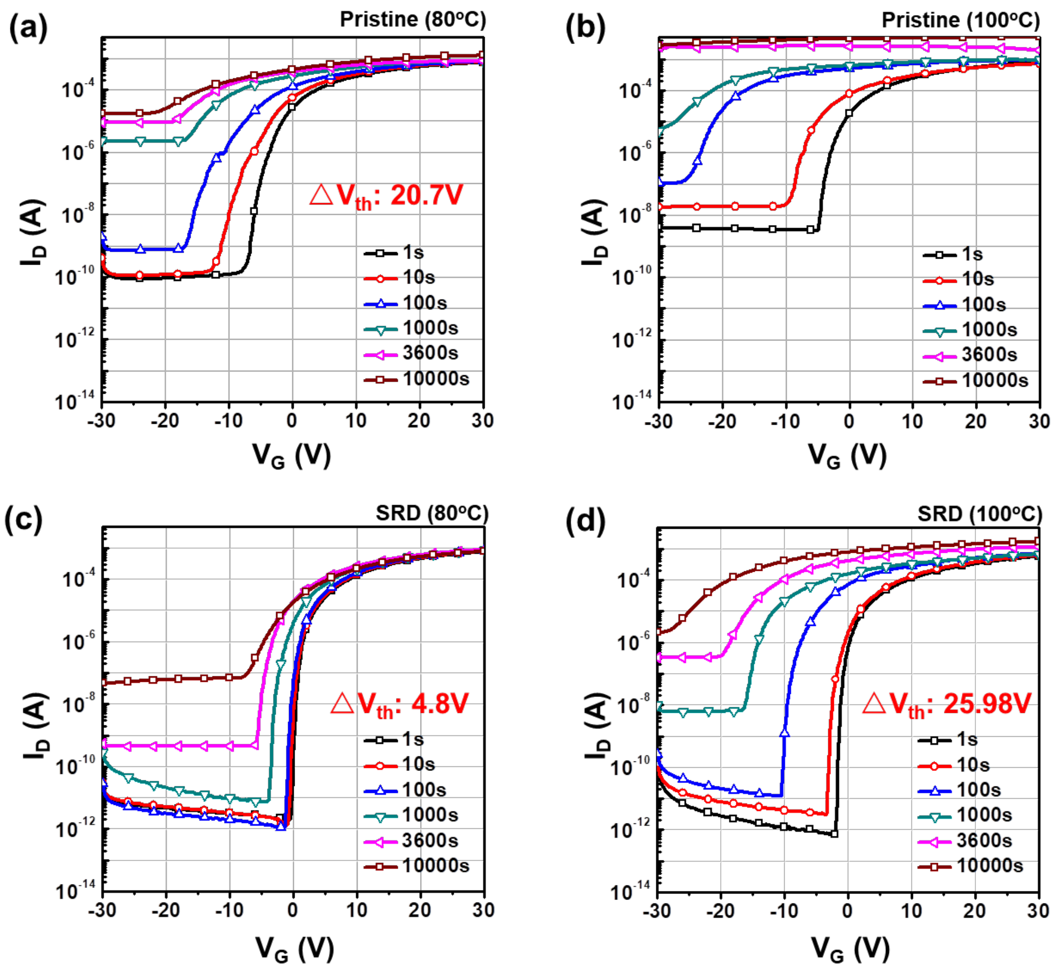

Figure S2. Variations of negative V_{th} shift under NBTIS with stress times for pristine (a) at 80°C and (b) at 100°C and SRD treated IGZO TFTs (c) at 80°C and (d) at 100°C

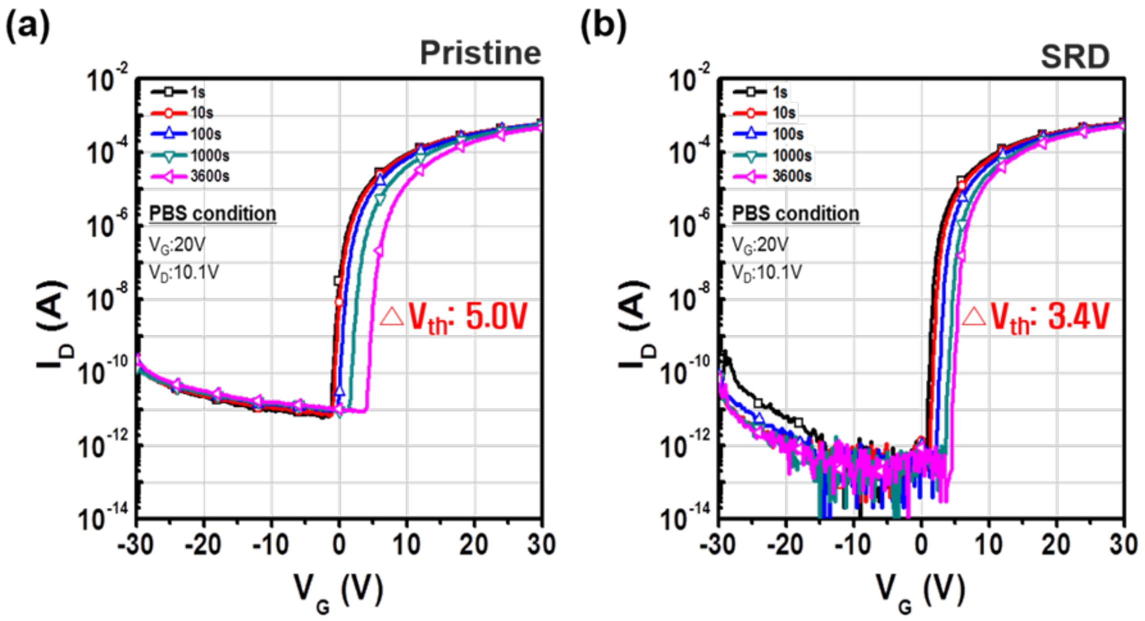

Figure S3. V_{th} of SRD a-IGZO TFTs under PBS with stress times: (a) Pristine and (b) SRD

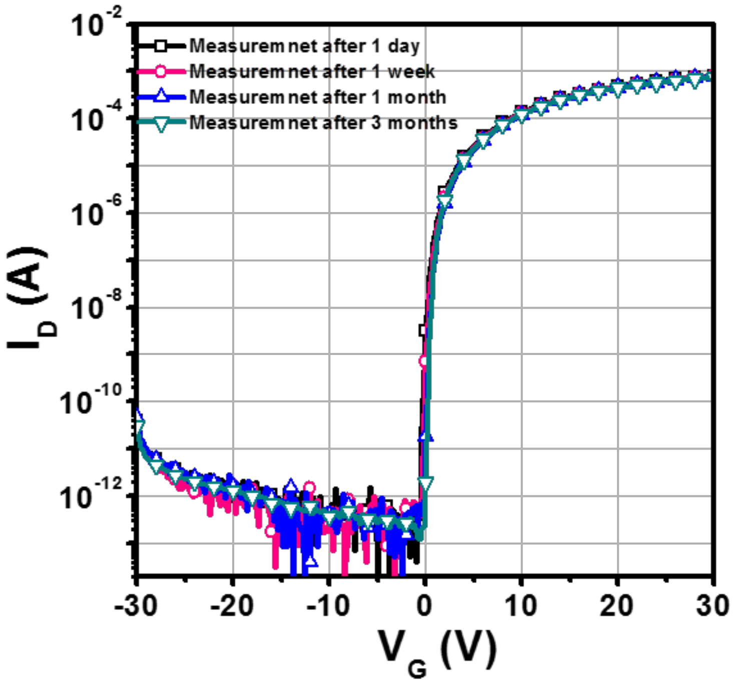

Figure S4. Transfer characteristics of SRD a-IGZO TFTs after 1 day, 1 week, 1 month, and 3 months

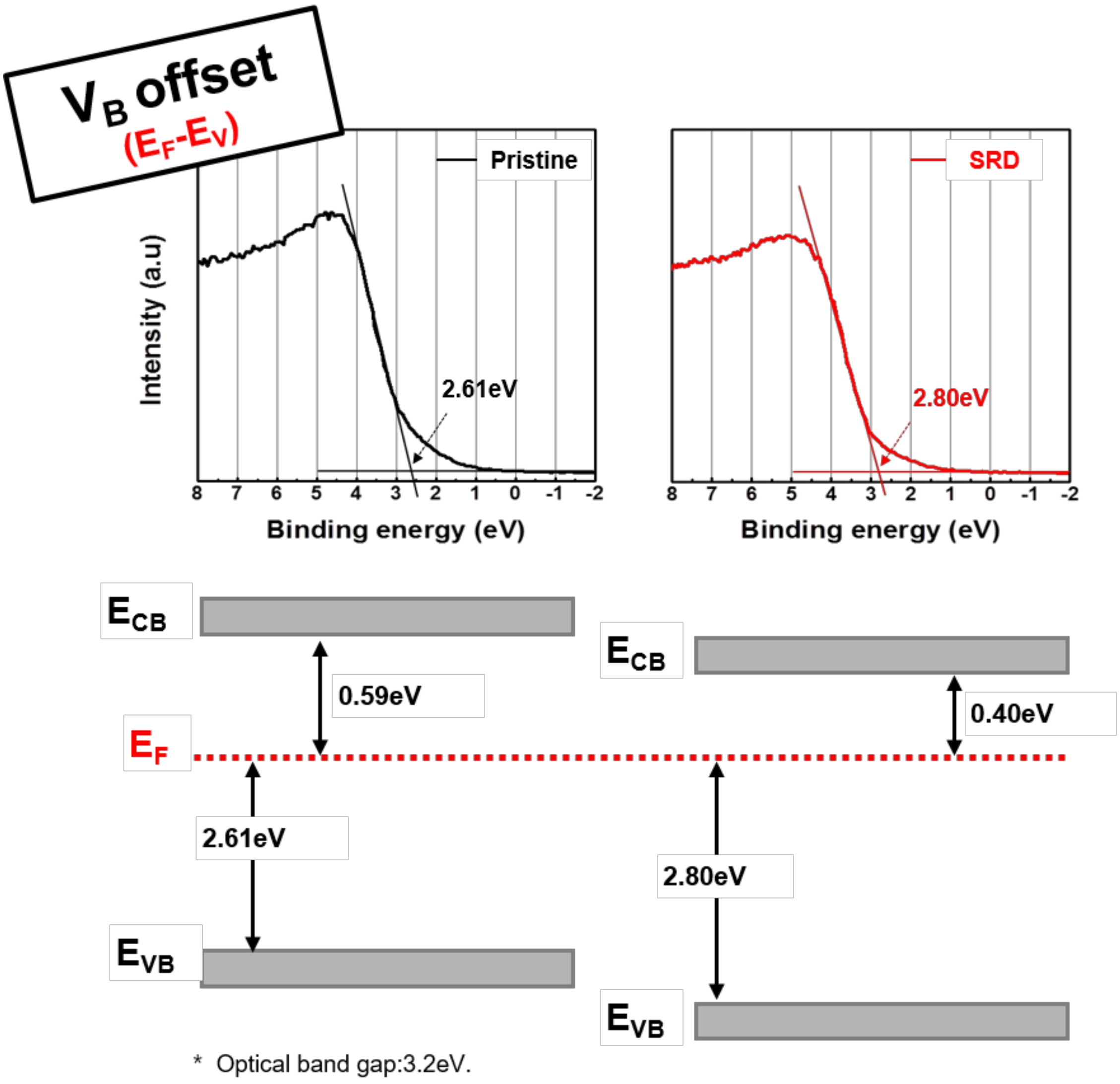

Figure S5. Variations of valence band offset spectra and band alignment for pristine and SRD a-IGZO TFTs

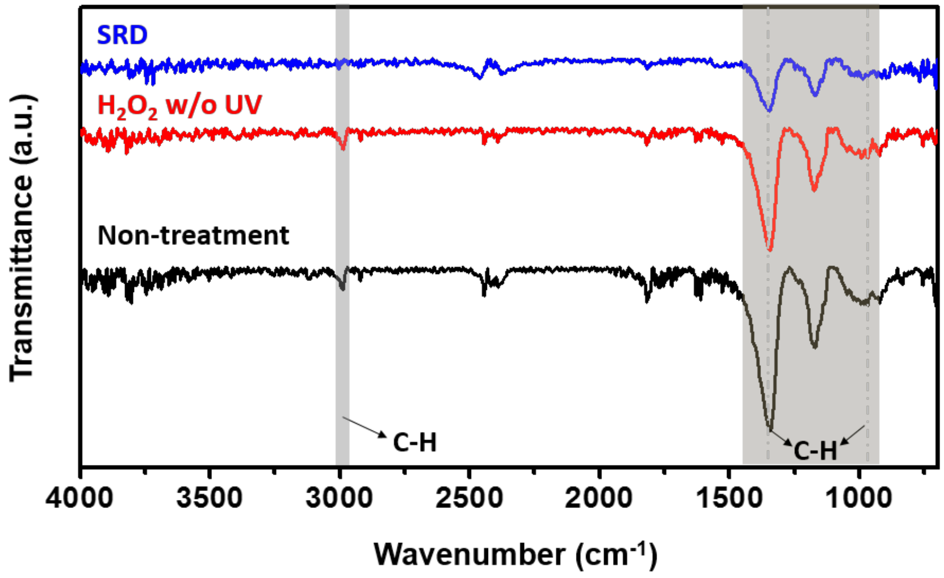

Figure S6. FTIR spectra of non-treatment, H_2O_2 w/o UV and SRD
